# Determinants and supporting factors for rebuilding nursing workforce in a post-disaster setting

**DOI:** 10.1186/s12913-019-4765-y

**Published:** 2019-11-29

**Authors:** Moe Hirohara, Akihiko Ozaki, Masaharu Tsubokura

**Affiliations:** 1 The Institute of Medical Care and Societal Health, 2-12-13-201 Takanawa, Minato-ku, Tokyo, Japan; 2 Research Center for Community Health, Minamisoma Municipal General Hospital, 2-54-6 Takami-cho, Minamisoma City, Fukushima Japan; 3 Department of Public Health, Fukushima Medical University School of Medicine, 1 Hikarigaoka, Fukushima City, Fukushima Japan

**Keywords:** Disaster management, Nursing workforce, Nuclear disaster, Great East Japan earthquake, The Fukushima Daiichi nuclear power plants

## Abstract

**Background:**

The workforce shortage is one of the major issues associated with the recovery of Minamisoma City in Fukushima Prefecture, after the Great East Japan Earthquake and the subsequent accidents at the Fukushima Daiichi Nuclear Power Plants in March 2011. While the radiation risks are often discussed as a major factor of evacuation, little is known about the actual reasons why the residents chose to evacuate, and what enables them to return.

This study aims to find the essential factors for rebuilding the workforce in a post-disaster setting by analysing the residents’ decisions about evacuation and the return to Minamisoma. In particular, we focus on the experiences of nurses as an example of healthcare workers, who play an important role in the disaster recovery.

**Methods:**

The data were obtained through qualitative interviews in a semi-structured form with 25 nurses from four hospitals in Minamisoma City. The interview questions focused on the reasons of their decisions on evacuation and return. The data were analysed by a thematic approach to investigate the major factors which led them to evacuate and enabled them to return afterwards, as well as the support they needed to resettle.

**Results:**

Nearly two-thirds of the interviewees chose to evacuate from Minamisoma with their family. Family conditions seem to be the predominant factor that influenced their decisions. In particular, having small children was a strong cause for evacuation. After a certain period of time, the nurses that evacuated were then faced with another decision about returning to the area; once again, having children, as well as other life factors, such as livelihoods, job opportunities and emotional attachment to the work, influenced this decision. On the other hand, radiation risk was a minor factor. Therefore, we analyse that improved support considering their life situations would contribute to the better retention of the nurses.

**Conclusions:**

We suggest measures such as parenting supports, ensuring job opportunities after return, and psychological support in the workplace as possible solutions for higher job retention.

## Background

The Great East Japan Earthquake occurred on March 2011, and the affected areas are still attempting to recover to this day. Depopulation is one of the major issues, as it deeply affects society and the economy. Higuchi et al. [1] found that the number of people who moved from disaster-stricken prefectures, such as Iwate, Miyagi, and Fukushima, increased by 30,799 in 2011. Particularly, Fukushima prefecture experienced a significant decrease in population, as 80% of the total emigration from the surveyed prefectures occurred here [1]. Moreover, the emigration of the younger population specifically increased because of the nuclear power plant accident following the earthquake in Minamisoma City (Ibid).

Minamisoma City is in a coastal area of Fukushima Prefecture. It was struck by a tsunami following the earthquake; over 4000 houses were damaged, which accounts for 18% of the total households in the most affected districts in Minamisoma [2]. Moreover, the tsunami caused the system to breakdown at the Fukushima Daiichi Nuclear Power Plant (NPP), which is located along the seashore, resulting in nuclear meltdowns and a series of explosions. After the first explosion occurred on 12 March, the Japanese government issued evacuation instructions to all areas within a 20 km radius of the NPP (Ibid). On 15 March, after there were several more explosions at NPP, the government issued a ‘stay-in-house’ order to all residents living within a 20-30 km radius of the NPP, which prevented logistics services from entering the area (Ibid).

In this period, hospitals in Minamisoma were confronted by the need of securing their staffs’ safety while they play an important role in maintaining the city functions and the population health. Table 1 is a timeline of the government’s evacuation orders and the operations of main hospitals in Minamisoma based on a survey by Rebuild Japan Initiative Foundation [3]. Among the four surveyed hospitals, a general municipal hospital run by the city municipality (hereafter Public Hospital), and two private hospitals (Private Hospital 1 and 2) are located within a 30 km radius of NPP. Another hospital, run by the Japan Agricultural Cooperatives (JA Hospital), was located outside of this area. The three hospitals located within the 30 km radius decided to transport their patients to other hospitals outside of the radius. Responding to another NPP explosion, Public Hospital and Private Hospital 2 advised the staff that those who would like to evacuate should leave at their own discretion. Moreover, although it was outside of the 30 km radius, JA Hospital also advised their staff to consider evacuation after it served as a transit point for patient transportation. In contrast, Private Hospital 1 did not mention staff evacuation as it continued its operation. After transportation was complete, and the patient wards were closed, many staff members including doctors, nurses, and clerical workers, faced the decision of whether to evacuate or remain at work.

**Table 1:** Timeline of events following the earthquake

Dates	Accidents / Government’s response	Hospitals’ operations
11 March 2011	The earthquake and tsunami occurred	Response to emergency patients
	Evacuation order for 3 km radius of NPP issued	
12 March	The explosion of Unit 1 at NPP	Transportation of patients began
	Evacuation order of 20 km radius	Voluntary evacuation advice to staff (Public Hospital and Public Hospital 2)
14 March	The explosion of Unit 3	
15 March	The explosion of Unit 4	
	Stay-In-House order for 30 km radius issued	
17 March	Emergency evacuation plan issued	
	The city’s evacuation transportation for residents began	
18–21 March		Private Hospital 2 and JA hospital closure
		Public Hospital’s patient wards closed, operations limited to outpatients
		Private Hospital 1 closure *Reopened 4 April

Among the hospital staffs, the number of the nurses who evacuated was prominent. The assistant director of the Public Hospital recalled that many of them were ‘pulled by the family’s evacuation’ [3]. It is notable that the majority of the nurses were female and take on the roles of a wife, mother, or caregiver to elderly parents, who seemed to have had greater conflicts between family matters and work. A study found that adult women and their children are the least likely to return from the place they evacuated [4]. From their demographic study in post-disaster Minamisoma, Zhang et al. [4] argue that the depopulation and decline of the workforce are largely attributed to the radiation contamination, which is now a long-term problem for the city.

However, little is known about the actual reasons why they chose to evacuate, and what enables them to return to the city. In fact, some nurses remained in Minamisoma throughout the disaster period, and others returned after their temporary evacuation. Therefore, an analysis of the differences in their decisions (evacuate/remain/return) can be useful to regain the loss of human resources following a disaster.

This study seeks the essential factors for rebuilding the workforce in a post-disaster setting by analysing the residents’ decisions about evacuation and the return to Minamisoma. Particularly, we focus on the healthcare workers as it plays an important for the population health and the city recovery. Due to the limited time frame of the research, we focused on one job category and chose nursing workforce as its decrease was one of those the most prominent. According to Ochi et al. [5], the number of healthcare workers, including doctors, nurses, and clerks, sharply declined after the disaster. Even after 18 months, the number of workers remained at 85% of the pre-disaster figure. In particular, the nurses and clerks, most of whom were female, seemed more likely to leave the job, as they were concerned about the accident’s effect on their children [5]. While it is widely discussed that the radiation risks were the major reason for leaving their job, some studies indicate that they had other important concerns relating to their loved ones and work responsibilities [6]. However, there are few studies that focus on this point.

Therefore, qualitative research was conducted to examine the experiences of the nurses, who work at the hospitals in Minamisoma City at present. Although many of them experienced a temporary evacuation shortly after the disaster, they have returned to their workplaces. This study analyses the major factors which led them to evacuate and enabled them to return afterwards, as well as the support they needed to resettle. Shedding the light on their life after the evacuation and resettlement, it discusses possible measures to rebuild the nursing workforce in a post-disaster period.

## Methods

This research investigates a) the factors that led the nurses to evacuate and the difference from those who remained, b) what enabled them to return, and c) what support is needed in the work environment in the aftermath. To examine these questions, qualitative interviews were conducted with the nurses from four hospitals in Minamisoma from 17 to 28 May 2018. The interviews focused on nurses due to the limited time frame of the research. Ethics approval was granted by the committee of Minamisoma General Municipal Hospital. Ozaki and Tsubokura made primary initial contacts with the directors, assistant directors and nurse managers of hospitals in Minamisoma to obtain the consent of participation in the research. The interviewees were recruited via nursing managers in each hospital. The contact with those who had evacuated and no longer work in Minamisoma was not made. All interviews were conducted by Hirohara in Japanese. Interview rooms were arranged in the hospitals and there were only one interviewer (Hirohara) and one interviewee in each interview setting. The interviews lasted for 45 minutes to 1 hour. They took a semi-structured form with an interview guide of a series of open-ended questionsand additional questions depending on the interviewee’s responses [7]. The questions focused on the nurses’ experience during and after the disaster, such as whether they evacuated or not, the duration of the evacuation, and the reason for the return. Written consent was obtained with all interviewees prior to the interviews. The interviews were recorded as audio data based on their consent, except for several cases in which the interviewees did not agree to be recorded. The data were transcribed in Japanese by a transcription service agency first, then transcriptions were translated into English and anonymized by Hirohara. The interview data were analysed based on a thematic approach, reviewing whole data recursively to examine key themes and storyline relating to the research questions [8]. Following the thematic analysis process defined by Braun & Clarke [9], the authors reviewed the data with several steps; reading the overall transcription and developing initial ideas of the data features, extracting the data features and developing the potential themes, and reviewing the relations of the themes the data extracts. A qualitative research software Nvivo was employed for data extract. To protect the interviewees’ privacy, all the audio data were deleted after the data analysis because the recorded voice may be identifiable.

## Results

The data were collected from interviews with 25 nurses in total. In this article, codes are allocated to each hospital and each interviewee. Namely, for the 10 nurses from the Public Hospital, codes M-1 to M-10 are allocated. Seven nurses from Private Hospita 1are O-1 to O-7, 4 from Private Hospital 2 are W-1 to W-4, and 4 from JA Hospital are K-1 to K-4.

Many were on duty when the earthquake occurred on 11th March, then stayed overnight at the hospitals responding to emergency patients and reorganizing the disrupted hospital facilities.

Of the 25 interviewees (23 females and 2 males), 16 of them (14 females and 2 males) chose to evacuate to other areas in Fukushima or to other prefectures with their families. After the evacuation, which lasted from 2 weeks to 12 months, they returned to their hospitals in Minamisoma. The other 9 nurses have remained in their positions at the same hospital until present. As all the interviewees lived with their family, family conditions seem to be the predominant factor that influenced their decisions. In particular, having small children was a strong cause for evacuation. After a certain period of time, the nurses that evacuated were faced with another decision about returning to the area; once again, having children, as well as other life factors, influenced this decision. Several issues in the work environment following their return were also observed. In the following sections, their common experiences in each phase are examined.

### Primary decisions

Shortly after each hospital finished transporting patients to outside Minamisoma, the nurses faced the decision to evacuate or remain. In this primary phase, their major concern was the safety of their family. The key difference between those who chose to evacuate and those who remained seemed to be whether they had small children. Many of the nurses with small children worried about the radiation’s impact on their children immediately after the NPP accident. This concern was commonly understood in the workplace, as some nurses were advised to evacuate by their managers or colleagues:

‘I talked to the manager “What can I do with my work?”. Then she told me “Your children are small, so you can evacuate.”’ (Nurse M-6, Public Hospital)

However, the interviewees’ fears of the radiation were quickly eliminated when they acquired knowledge from the doctors and specialists in the hospitals. One nurse mentioned that the radiation issue became less important in his decision, after learning about the issue:

‘I knew some radiographers and asked them about it (the radiation risk) … I learned that it is not an urgent situation that we definitely have to evacuate … So, I thought I could remain’. (Nurse M-4, Public Hospital)

More importantly, some nurses with small children found other aspects in their living environment more challenging. One nurse recalls how she eventually chose to evacuate outside of the city with her children after hearing recommendations from others:

‘We took refuge to a gymnastic hall first, but the children were too noisy for other evacuees. So, we moved into a room in the hospital I work. But the food stocks were getting less. People around me told that “It is about time you evacuate”’. (Nurse M-4, Public Hospital)

The example above reveals the difficulties faced in the physical and social environments, such as the limited availability of food, infant milk, and diapers, or the attitudes of people who were over-concerned or intolerant of a mother with small children. Furthermore, the sudden life changes brought on by the disaster, such as the closure of the schools or loss of friends, impacted the children’s mental health. In one case, the nurse had to leave the hospital to sooth her daughter:

‘My children had already evacuated to Shirakawa (neighbouring town of Minamisoma) on the next day of the power plant’s accident … And the reason I decided to leave was … my daughter had lost a friend because of the tsunami … she was very upset. We were living separately, but she cried “Mom, come back home”. So I had to go to her. And I had to leave the hospital although I didn’t want to … ’ (Nurse O-2, Private Hospital 1)

As in this example, some nurses had to travel between their work and their family to care for the children’s mental health. In cases where travelling was too difficult to sustain due to distance or other issues, they tended to leave their job. On the other hand, most of the nurses who remained at work were without children, or their children were mature enough, so that they were able to stay in Minamisoma with their partners or just by themselves. One nurse described how she prioritized work at the hospital while her children evacuated:

‘My daughter was already married, and my son was a high school student. So, they were not like small children … I told them “You can eat your foods by yourself. But there are patients who cannot eat by themselves at the hospital. Mom is not going anywhere like a battlefield. The hospital is safe. So, you should go ahead and evacuate”’. (Nurse O-7, Private Hospital 1)

From these findings, having small children was the dominant factor in the primary decisions. Moreover, access to daily supplies, social environments, and the mental health of their children was found to be major determinants that influenced their choice to evacuate. Furthermore, it was found that the health risks associated with the radiation were worrisome in the earlier portion of this phase; however, it was less prominent later on. Notable differences in decision making between males and females were not observed for several reasons. Firstly, there were only two male interviewees who could participate in this research, which may not make an adequate comparison to 23 female interviewees. Secondly, although both male interviewees remained in the city, this was not because of gender. One commented he needed to take care of the people who took refuge near the hospital, and the other mentioned he was concerned for the patients at the hospital, and similar responses were made by other female interviewees. Above all, not having small children seemed to be the primary factor of their decision like other female nurses.

### Reasons for return

The evacuation period for the interviewees ranged from 2 weeks to 1 year. In this mid−/long- term perspective of the aftermath, decisions to return to Minamisoma were based on concern for their livelihoods and for their children’s education, as well as attachment to familiar workplaces. On the other hand, the radiation issue was no longer mentioned as a major concern. The interview results also found some issues in the work environments during the post-disaster period.

### Work opportunities

The primary factor for the decision to return was work opportunities. As the workers became concerned about their livelihoods after the evacuation, they sought to work again. The nurses’ actions in this situation mainly fell into two categories: finding a job in their evacuation destinations or returning to their former jobs in Minamisoma. The decisions were largely based on where they could obtain a job more quickly, as well as information on the recovery situation of their former workplaces. For example, one nurse explained that she started a new job after evacuation because the hospital in Minamisoma was still closed:

‘The hospital did not inform me directly, but I often asked my fellow nurses “How is the hospital?”, because I was concerned … I am a single mother, so I thought I cannot live without earning money. So, I called the hospital director and got permission to work elsewhere … I worked in Yamagata (a prefecture she evacuated) for one year’. (Nurse W-2, Private Hospital 2)

On the other hand, a nurse from another private hospital revealed that she came back to the city to work again, as she could not find a job where she evacuated:

‘I looked for what I can do in Tochigi (a prefecture she evacuated), but I couldn’t find. So, I came back … the hospital had been closed just for a week, but it started again in April, with the staffs who remained there’. (Nurse O-1, Private Hospital 1)

The crucial difference between the two examples was the timing of the hospital’s reopening. Private Hospital from the latter example had reopened in early April, while Private Hospital 2 did not have a clear plan for their reopening at that time. More importantly, Private Hospital 1 informed others of the hospital’s situation directly, as the head nurse frequently contacted the evacuated nurses by phone, letter, and sometimes personal visits. Such attempts were characteristic of Private Hospital 1 and contributed to reducing uncertainty about work conditions after returning. On the other hand, some nurses from other hospitals indicated that there was a lack of communication from their workplaces:

‘Maybe I should have called them, but my managers also did not phone me while I was on a leave … It was better if they could contact and tell me about the hospital’s situation and ask me about my situation’. (Nurse M-3, Public Hospital)

Moreover, delayed or uncertain information about the hospitals’ reopening seemed to hinder the return of the nurses, and led them to consider resigning in some cases:

‘My former colleague told me “You can transfer to here if you don’t have a job”. So, I was thinking I could go to work somewhere new because I did not know when will the hospital in Minamisoma restart the operation’. (Nurse K-3, JA Hospital)

Considering these examples, the guarantee of job opportunities upon returning was a crucial condition. Direct communication from the hospital was also imperative to reduce the barriers for nurses to return.

### Family conditions and children’s education

Another important reason for returning was related to the family’s conditions. For instance, in cases where their partners or parents had a job or owned business in Minamisoma, the nurses tended to consider returning. In addition to these conditions, education for their children was a prominent factor in deciding when to return. For example, one nurse revealed the reason for her return after evacuating and working for a year in a new location:

‘My younger son was about to enter a junior high school. He could go to a school in Yamagata (where she evacuated). But, if so, we might have to live there for a long time. My father had already returned by himself for his business … So, his entering school was a good occasion to discuss, and we decided to return’. (Nurse W-3, Private Hospital 2)

As shown above, enrolling their children in further education, mainly in junior high or high school, played a major role in deciding when to return. The location of their school can largely influence their life for subsequent years, as it impacts where the children will go to college or find a job. Additionally, the children wished to reunite with their friends after their school reopened. Therefore, many nurses thought it was better to return to their hometown for their children. However, parenting in post-disaster Minamisoma was not easy for working mothers; for example, some schools required parents to transport their children to and from school. One nurse recalled that some of her colleagues did not return because the education environment outside of Minamisoma was more preferable:

‘As I was working, my husband’s mother could collect my children from school. But, I don’t know if it was good for the children … I heard some people did not come back because there were more schools and more students, and the foundation for education was more equipped in other places’. (Nurse M-2, Public Hospital)

This comment also indicates that it was essential to get other family members’ support to take care of the children. Otherwise, it might be too difficult for them to continue.

### Work for mental recovery

The nurses’ will for returning to a familiar workplace was another determinant. Many of them mentioned an attachment to their hometown, hospitals, and colleagues, as well as a responsibility for the patients. Continuing the work seemed helpful not only for their livelihoods but also for their mental recovery:

‘The work was the only thing which had not changed since before the disaster. If I did not continue this, I could not keep up myself … Because everything turned into a blank, and I might have been lost wondering how I was going to live’. (Nurse K-4, JA Hospital)

As this example shows, engaging in regular work helped them to recover from the shock and depression that followed the disaster. Therefore, many of them were in favour of getting back to work rather than being reluctant.

### Issues in the work environments

However, there were some cases in which nurses experienced increased stress following their return. Firstly, there was a certain feeling among the returnees, often referred to as ‘*Oime’* in Japanese; this can be described as a sense of guilt from having left the hospitals. One nurse recalled that she felt undeserving of a promotion to a manager position because she had evacuated, and she was criticized when she came back.

‘For a while, there was an atmosphere indicating that those who had evacuated were kind of guilty … Some even said to me “With what face you came back?”’ (Nurse M-4, Public Hospital)

Such feelings were not only caused by direct criticism, as another nurse recalled the feeling of *Oime* as she was concerned for the other nurses who had remained:

‘I cannot deny that I felt *Oime* because some other nurses had been enduring since after the disaster … This feeling just has been within me’. (Nurse M-8, Public hospital)

In most cases, such discomfort seems to have been caused simply by a lack of proper communication rather than an intention to blame the returnees personally. A nurse that remained in place during evacuation recalls the workplace atmosphere:

‘There was a kind of a wall between those who could have the same life as one before the disaster and those could not. So, we could not have a heart-to-heart talk, and did not care enough for the others, being occupied with one’s own businesses. (Nurse M-6, Public Hospital)

Considering these comments, both the nurses that remained in place and those that evacuated then returned felt uneasy to express their everyday-life concerns because of the differences in their experiences. This lack of communication caused discomfort in the workplace for a certain period of time. Furthermore, there were several cases in which nurses had mental breakdowns due to work. It was especially prominent among nurses who were managers, as they had to cope with the scarcity of human resources and increased needs from patients. One nurse revealed that she became incapable of work at one time:

‘When I was a manager in an outpatient ward, I became mentally unstable … there was a period that the number of outpatients increased suddenly. The number of staffs was scarce, so I felt hard being a manager. I was urged by myself thinking I have to do this, and I have to do that … ’ (Nurse O-1, Private Hospital 1)

Due to a strong sense of responsibility, the nurse became overwhelmed by her duties. Eventually, she became unable to leave her house. However, she decided to return to work after she consulted with the head nurse:

‘I took a leave for some months. Then, the head nurse asked me “Do you want to move to long-term care wards?”. So, I decided to move to the other ward, being removed from the manager position of the outpatient ward. Then it has been stable until now’. (Nurse O-1, Private hospital 1)

This example indicates that coordination with a supervisor can often prevent nurses from leaving their job, thus contributing to worker retention.

The interview results revealed that the nurses made spontaneous decisions to return when the conditions were suitable for their life situations, especially concerning livelihoods and children’s education. However, there were many cases in which they delayed their decision because of uncertainty in terms of job opportunities or a lack of support for parenting. Also, the work environment after returning was stressful, and it sometimes resulted in them leaving their job. From these findings, it can be said that improved support regarding parenting, job opportunities, and work environment might improve the retention of the nurses in a post-disaster setting. In the following chapter, while summarising the essences of the obstacles, possible support measures to keep the nurses in the affected areas and the health facilities located there will be discussed.

## Discussion

### Adequate understanding of the radiation risk

Before we discuss the major challenges and possible solutions for the return of the nurses, it is important to note that the radiation risk was not a critical threat for most of the interviewees. Although there was a certain level of concern in the early phase of the aftermath, it could be seen that the nurses quickly resolved their fear of the radiation as they gained adequate knowledge from the doctors and specialists in the hospitals; studies confirm that the radiation risk was not as great as was originally perceived. For example, according to the screening conducted by Kodama et al. [6], three to 4 months after the disaster, the internal radiation exposure among the public hospital staff was far below the level which causes health problems. This tendency may be characteristic among only nurses, as some other studies pointed out that concern for the radiation risk among the overall population in Fukushima remained strong. For instance, Orita et al. [10] demonstrated the survey results indicating that half of the surveyed residents in Fukushima remained anxious about the health effects of the radiation on children 3 years after the NPP accident.

Such differences between the perceptions of the general population and the interviewees may come from the access to adequate information, as the interviewees work in hospitals and have greater access to expert knowledge on radiation risks. One study focusing on the difference in radiation risk perception between the general population and the expert group (employees of the nuclear research centre) analysed the main reasons for the lower risk perception among the expert group. The main reasons were presumably because of more accurate knowledge and familiarity with the concept of radioactivity [11]. Moreover, Hasegawa argues that inadequate information about nuclear accidents and associated radiation risks from public authorities hindered the evacuees’ decisions to return to a large extent [12]. Therefore, it can be said that if accurate information on radiation risks was provided by authorities, fear of the radiation among the general evacuees could be relieved at an earlier time. More importantly, Sato and Lyamzina suggest that more research is needed on the indirect consequences of the nuclear accident on the livelihoods and at the community level as it pertains to the long-term recovery policy [13]. Such aspects are discussed in the following sections.

### Support for parenting

The living environment in post-disaster Minamisoma presented several issues in terms of parenting. Firstly, the material and social environments were challenging, as there was limited access to supplies for infants and many schools were closed. Secondly, disrupted living environments made it difficult for the mothers to work while caring for the children. Moreover, family separation caused other issues, as many nurses experienced separation either from children or partners. Such situations caused further issues for the families, such as emotional insecurity for the children (seen in the results in section 3–1), or deteriorated relationships with partners [14]; these situations make parenting even more difficult.

Difficult parenting environments can also cause mental issues, as a study by Goto et al. [15] revealed that the mothers in *Soso* District, which includes Minamisoma and several neighbouring towns, displayed symptoms of depression; such issues are common in many post-disaster settings. For example, after hurricane Katrina in 2005, mothers in the affected area suffered from a lack of childcare assistance due to the loss of family/relative support networks, and became overwhelmed by the needs of caring for their children and elderly parents at the same time [16]. In such situations, some voluntary programmes offering daycare for children were found to be very helpful among the parents, as private childcare services were filled due to high demand (Ibid). Therefore, the enrichment of childcare support can be key to retaining nurses with children. For example, Minamisoma Municipal General Hospital opened a nursery centre inside the hospital in April 2018 to support the hospital staff with small children [17]. This, however, was only the case in a public hospital, and it might be difficult to establish a new daycare facility in smaller hospitals. Therefore, other support systems, such as coordinating work schedules through part-time or day-time shifts should be considered as well.

Understandably, not all the evacuees would be convinced to return based solely on parenting support, as some decided to settle in a new place for educational purposes, and such decisions should be made based on the individual’s choice. Nevertheless, coordinating the nurses’ schedules with consideration for their personal situations can be encouraging for those who struggle to resettle themselves in post-disaster situations while they wish to work.

### Ensuring job opportunities

Among those who evacuated, regaining their livelihoods after losing their houses or leaving jobs was a crucial problem. This issue was common among the evacuees of different occupations, as statistics revealed that 40% of the evacuees from disaster-stricken prefectures, including Fukushima, remained unemployed for 1 year after the disaster (Asahi Shimbun, 2012 cited by [12]). Finding a job in a new location may prove difficult, as some employers are not willing to hire the evacuees due to the uncertainty associated with how long they will remain [14]. However, as the research results indicate, returning to their hometown was daunting as it was uncertain if there were job opportunities. This uncertainty became a major obstacle in their return. Therefore, ensuring job opportunities is essential to retain workers. To achieve this, management should provide explicit information about the recovery plan and communicate frequently with workers about when they can return to their positions. For example, head nurses or nurse managers could play an important role in communicating with evacuated employees. Moreover, it is important to ensure financial support for returning evacuees. Fukushima Prefecture has been employing different measures to tackle the healthcare workers shortages, such as financial support for those who move to Minamisoma from other areas (Fukushima [18]), or enrichment of education and scholarships for the nurses [19]. However, the nurses who did not evacuate may not feel that they are receiving enough support. A survey by Sato [20], conducted with nurses along the seashore area of Fukushima 5 years after the disaster, revealed that many of them were not satisfied with work conditions such as workload and salary. Therefore, there is a need to clarify the gap between the necessary support programmes and the present systems and ensure that the nurses who did not evacuate utilize those support programmes.

These arguments may not be applicable to all cases. For example, a partner’s job has a substantial impact on where one chooses to live, especially for married female nurses. Thus, when the husband has found a job in their new location, the nurses tend to prioritize the husband’s job and look for a job in their new location. Therefore, it is important that hospitals contact the evacuated staff and confirm their will to return, regardless of their decisions.

### Psychological supports at workplaces

The results of the interviews also indicate that the nurses were in need of psychological support for several different issues, such as discomfort among colleagues and pressure stemming from resource scarcity. Nurses tend to experience greater stress in post-disaster settings, as they are caregivers responding to the aftermath and disaster victims at the same time [21]. It was also observed from the interviews that the nurses felt uneasy sharing their concerns with colleagues, as there was an emotional barrier between the nurses that remained and the returnees. Such situations may prolong the heightened stress levels. A study confirms that the nurses in Fukushima still have greater stress levels than the general population after 4 years of the disaster, and their conditions were considerably affected by burdens related to work and daily life (Ibid). To address this situation, nurses need support to help them cope. Particularly, a survey by Nukui et al. [22] found that many nurses mentioned sharing mental distress with colleagues and supervisors as a major obstacle to overcome. Therefore, close communication must be established between the supervisors and colleagues to enable nurses to share their daily concerns. Moreover, the example of the nurse who had returned from a mental breakdown shows that it is helpful to communicate and change job positions when necessary if nurses are having difficulties coping with their tasks.

Mental health is indeed highly dependent on the individual, and some people would be more traumatized by the experience of the disaster than others; thus, some may simply prefer not to return. Again, the individual choice should be respected first under such conditions. However, one study suggests that mental resilience is formed not only by personal traits; it found a significant association between mental resilience and sociodemographic factors, such as employment or healthy lifestyles, which possibly reduce the risk of PTSD or other mental illnesses [23]. Further research is needed to examine the possible roles that workplaces can perform to reduce stress in the post-disaster setting.

## Conclusion

This study focused on the experiences of the nurses in Minamisoma in the middle of the aftermath and their return to work in the post-disaster period. The findings indicated that major concerns were closely related to their everyday life, especially pertaining to living environments for children (including material, social, and mental aspects) and livelihood, rather than fear of radiation. Moreover, we found that stronger support is needed in the areas of parenting, job opportunities, and work stress to help the nurses continue to work after their return. Such support in the aftermath could improve the retention of nurses in Minamisoma, and in other similar settings. It should be noted that the interviews were conducted only with those who currently work in Minamisoma, and we did not contact those who had resigned; therefore, the findings do not cover every aspect of the decision to stay in a new location after evacuation. Additionally, tendencies may be different for other groups with wider varaiety of gender/age/occupation. Therefore, the results may not be generalized for other workforces. Further research is needed to apply the results to a wider context and examine the factors associated with job retention.

## Supplementary information

**Additional file 1.** Interview guide, Interviewguide.docx, English interview guide translated by the author.

## Data Availability

Not applicable.

## Abbreviations

NPP: the Fukushima Daiichi Nuclear Power Plant
