# Supplementary material for: Determinants and supporting factors for rebuilding nursing workforce in a post-disaster setting
Source: BMC Health Serv Res. 2019 Nov 29;19:917. doi: 10.1186/s12913-019-4765-y (PMC6884864; doi:10.1186/s12913-019-4765-y)
Supplement: Supplementary file 1 — Additional file 1. Interview guide, Interviewguide.docx, English interview guide translated by the author. [file 12913_2019_4765_MOESM1_ESM.docx]

**Cover Letter**

Maria Zalm, BioMed Central

Editor

BMC Health Services Research

Address: BioMed Central

The Campus, 4 Crinan Street

London N1 9XW

United Kingdom

Submission Date: 20^th^ January, 2019

Dear Ms. Zalm,

I am writing to submit our manuscript entitled, " Determinants and supporting factors for retaining medical workforce in a post-disaster setting " for consideration as a research article of BMC Health Services Research. We examined the factors of the retention of a medical workforce in the aftermath by analysing the experiences of the nurses after the Great East Japan Earthquake disaster. We found that many nurses chose to evacuate from the area and to return afterward, based on their children’s living conditions and livelihoods, while radiation risk was a minor factor. Our findings suggest measures such as parenting supports, ensuring work opportunities after return, and psychological support in the workplace as possible solutions for higher job retention.

Given that workforce shortages are major issues in health services in post-disaster settings, we believe that the findings presented in our paper will appeal to the health service professionals/researchers who subscribe to BMC Health Services Research. Our findings will allow your readers further discoveries on the allocation of healthcare resources, especially human resources.

This manuscript expands on the prior research ”Hospital staff shortage after the 2011 triple disaster in Fukushima, Japan-an earthquake, tsunamis, and nuclear power plant accident: a case of the Soso District” conducted and published by Ochi and the colleagues in Plos ONE, in October 2016. Also, it examines a different aspects of the issues explored in the following papers.

- “Impact of natural disaster combined with nuclear power plant accidents on local medical services: a case study of Minamisoma Municipal General Hospital after the Great East Japan Earthquake” by Kodama, Y., Oikawa, T., Hayashi, K., Takano, M., Nagano, M., Onoda, K., Yoshida, T., Takada, A., Hanai, T., Shimada, S. and Shimada, S., published in Disaster medicine and public health preparedness, December 2014
- ”Mental health and related factors of hospital nurses: an investigation conducted 4 years after the Fukushima disaster” by Nukui, H., Murakami, M., Midorikawa, S., Suenaga, M., Rokkaku, Y., Yabe, H. and Ohtsuru, A., published in Asia Pacific Journal of Public Health vol 29, March 2017
- “Disappearing everyday materials: The displacement of medical resources following disaster in Fukushima, Japan” by Abeysinghe, S., Leppold, C., Ozaki, A., Morita, M. and Tsubokura, M., published in Social Science & Medicine, 191, October 2017

Each of the authors confirms that this manuscript has not been previously published and is not currently under consideration by any other journal. Additionally, all of the authors have approved the contents of this paper and have agreed to the BMC Health Services Research's submission policies.

Should you select our manuscript for peer review, we would like to suggest the following potential reviewers because they would have the requisite background to evaluate our findings and interpretation objectively.

- Hiroshi Nukui, Department of Radiation Health Management, Fukushima Medical University, Fukushima 960-1295, Japan
- Turner SB, University of Alabama, Capstone College of Nursing, Tuscaloosa, Alabama.
- Emiko Konishi, Kagoshima University Faculty of Medicine, , Kagoshima, Japan, [emikok88@yahoo.co.jp](mailto:emikok88@yahoo.co.jp)

To the best of our knowledge, none of the above-suggested persons have any conflict of interest, financial or otherwise.

Each named author has substantially contributed to conducting the underlying research and drafting this manuscript. Additionally, to the best of our knowledge, the named authors have no conflict of interest, financial or otherwise.
 Also, the authors confirm that the individuals who contributed to the research agreed to include their names in the acknowledgements.

Sincerely,

Moe Hirohara

Corresponding Author

Visiting Researcher

The Institute of Medical Care and Societal Health

2-12-13-201 Takanawa, Minato-ku, Tokyo, JAPAN, 108-0074

moehirohara@gmail.com

Tel: +81 45 813 0597

Fax: +81 45 813 0597
